# Suitable binder for Li-ion battery anode produced from rice husk

**DOI:** 10.1038/s41598-021-95297-9

**Published:** 2021-08-04

**Authors:** Seiji Kumagai, Yusuke Abe, Masahiro Tomioka, Mahmudul Kabir

**Affiliations:** 1 Department of Mathematical Science and Electrical-Electronic-Computer Engineering, Akita University, Tegatagakuen-machi 1-1, Akita, 010-8502 Japan; 2 Joint Research Center for Electric Architecture, Akita University, Tegatagakuen-machi 1-1, Akita, 010-8502 Japan

**Keywords:** Materials for energy and catalysis, Batteries, Batteries

## Abstract

Rice husk (RH) is a globally abundant and sustainable bioresource composed of lignocellulose and inorganic components, the majority of which consist of silicon oxides (approximately 20% w/w in dried RH). In this work, a RH-derived C/SiO_x_ composite (RHC) was prepared by carbonization at 1000 °C for use in Li-ion battery anodes. To find a suitable binder for RHC, the RHC-based electrodes were fabricated using two different contemporary aqueous binders: polyacrylic acid (PAA) and a combination of carboxymethyl cellulose and styrene butadiene rubber (CMC/SBR). The rate and cycling performances of the RHC electrodes with respect to the insertion/extraction of Li ions were evaluated in a half-cell configuration. The cell was shorted for 24 h to completely lithiate the RHC. Impedance analysis was conducted to identify the source of the increase in the resistance of the RHC electrodes. The RHC electrode fabricated using PAA exhibited higher specific capacity for Li-ion extraction during the cycling test. The PAA binder strengthened the electrode and alleviated the increase in electrode resistance caused by the formation of the interphase film. The high affinity of PAA for SiO_x_ in RHC was responsible for the stabilization of the anodic performance of Li-ion batteries.

## Introduction

Li-ion batteries (LIBs) are widely used to store energy for mobile devices such as smart phones, tablets, and laptop computers, because they possess high energy densities and excellent cycling characteristics. LIBs are also preferred over conventional lead-acid and nickel-hydrogen secondary batteries for manufacturing high-capacity energy-storage systems used in electric vehicles and to stabilize the output of renewable energy generators^1^. To meet the industrial and social requirements for increased energy density and lifetime from LIBs, much effort has been devoted to cell design and assembly, and to the development of electrode active materials, electrolytes, and separators. Graphite has been used for many years as an anodic active material for LIBs because of its low cost, abundance, low and flat potential profile during Li-ion insertion and extraction, and familiarity. Recently, silicon (Si) has attracted much attention owing to its very high theoretical specific capacity for Li-ion insertion and extraction^2^. The theoretical specific capacity of Si is 4200 mAh g^–1^ in the form of Li_4.4_Si, or 3600 mAh g^–1^ in the form of Li_3.75_Si, which remains stable at room temperature. The specific capacity of 3600 mAh g^–1^ is approximately ten times as high as that of graphite (372 mAh g^–1^). However, the severe volumetric expansion of Si (approximately 400%) during the Li-ion insertion-extraction process can destroy the structure of the anode, leading to the loss of electrical contact. This increases the impedance, which rapidly degrades the capacity of Li-ion cells^3,4^. Substantial countermeasures are required to overcome this drawback of Si to realize its usage in next-generation energy storage systems.

The first countermeasure is to use a mixture of Si and carbon (graphite or hard carbon)^5–16^, which can alleviate the negative effect of Si on the LIB anode. The second countermeasure is to use silicon oxides (SiO_x_; 0 ≤ x ≤ 2). Lithium silicates (Li_x_SiO_y_) or lithium oxide (Li_2_O) formed by the reduction of SiO_x_ can mitigate the expansion^17^. The theoretical specific capacities of SiO_2_ and SiO have been reported to be 1965 mAh g^–1^ and 2043 mAh g^–1^^18,19^, respectively, which were calculated considering that the Si in SiO_x_ can be reduced to pure Si. Despite its much lower specific capacity for Li-ion insertion and extraction than that of pure Si, the combination of carbon and SiO_x_ (C/SiO_x_ composite) has garnered much attention^20–24^. C/SiO_x_ composites have higher specific capacities than that of graphite, and are expected to possess high rate-capabilities and long cycle-lives^25^. The third countermeasure is to use binders that can minimize the negative effects of the expansion of Si and SiO_x_^17,26^.

The primary role of the electrode binder in LIB cells is to enhance the mechanical strength of the connection between the solid components (active material and conductive agent) and the current collector to maintain electrical connectivity to facilitate electrochemical reactions. The traditional, non-aqueous polyvinylidene fluoride (PVDF) binder cannot reliably withstand the change in volume of Si or SiO_x_ particles during the uptake and release of Li ions because of the weak Van der Waals force between the binder and the active material. Currently, aqueous binders are generally used to fabricate LIB anodes. Polyacrylic acid (PAA), carboxymethyl cellulose, and combinations of carboxymethyl cellulose and styrene butadiene rubber (CMC/SBR) are typical aqueous binders^27–35^. Hydrogen bonds with hydroxyl groups (–OH) or carboxyl groups (–COOH) can produce strong adhesion between Si-based particles, thereby improving the electrochemical performance^36,37^.

Carbon/silicon oxide (C/SiO_x_) composites can be readily obtained by carbonizing rice husk (RH), which is an agricultural waste^38,39^. The major components of RH are organic materials such as hemicellulose, cellulose, and lignin (approximately 80% w/w), and minerals such as silicates, phosphates, and potassium and sodium salts, which are derived from paddy soil. Rice is a silicicolous plant that requires the uptake of silicic acids for its growth and accumulates them in its stems and grain husks. More than 90% (w/w) of the minerals in RH are generally composed of SiO_x_ (x was mainly at 1–2)^40^. The annual global production of paddy rice is in excess of 715 million metric tons^41^, indicating that approximately 180 million metric tons of RH are produced every year as an agricultural byproduct. Owing to its abundant and stable annual production, the electrochemical performance of carbonized RH with respect to the uptake and release of Li ions has been investigated to effectively utilize it and conserve fossil fuels^42–45^. However, the aforementioned studies used excessive amounts of the binder and conductive agent (≥ 30% w/w) for electrode preparation, which is impracticable for the manufacture of LIB cells. In addition to this, the suitability of the binder for RH-derived C/SiO_x_ composite (RHC) has not been verified.

For the sustainable development of global industry and agriculture, there is a strong drive to replace fossil resources for the graphite used in LIBs using a sustainable and abundant bioresource. In the present study, we fabricated electrodes using RHC as the active material. Two species of aqueous binders, PAA and CMC/SBR, were used to fabricate the electrodes to compare their suitability for binding RHC intended for use in LIB anodes. Finally, the Li-ion insertion and extraction properties of the fabricated electrodes and their cycling stabilities were evaluated to gain insight into the type of binder that is suitable for fabricating electrodes using RHC as the active material.

## Results and discussion

### Material properties of RHC and RHC electrodes

**Figure 1:**
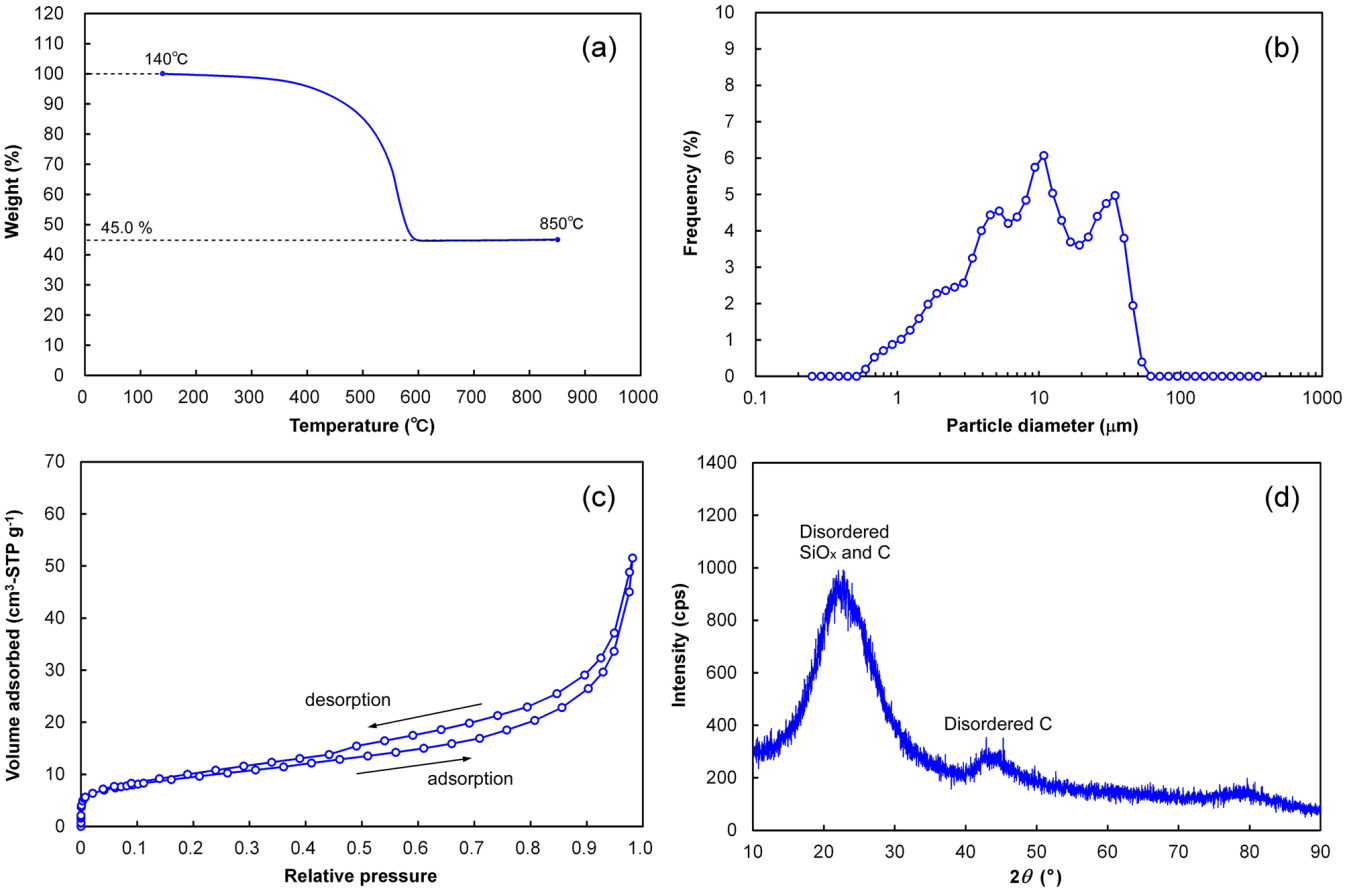
Material properties of powdered RHC. (**a**) Thermogravimetric response in air at the heating rate of 10 °C min^–1^, (**b**) particle size distribution, (**c**) nitrogen adsorption–desorption isotherm at – 196 °C, (**d**) X-ray diffraction pattern.

The material properties of RHC were evaluated by thermogravimetric, particle size, and gas-adsorption analyzers and are shown in Fig. 1. The weight of RHC in air under increasing temperature decreased up to 600 °C due to combustion in air and thereafter remained constant up to 850 °C. The SiO_x_ content was calculated to be 45.0% (w/w) based on the loss in weight between 140 and 850 °C. The particle size of RHC was distributed from 0.6 to 50 μm, with the mean diameter of the particles being 9 μm. The nitrogen adsorption–desorption isotherm of RHC exhibited a sharp increase at relative pressures lower than 0.02, a gradual increase at relative pressures exceeding 0.02, and a hysteresis loop in the high-relative-pressure region. The isotherm obtained can be categorized as IUPAC type IV^46^, indicating the existence of micropores and mesopores in RHC. The Brunauer–Emmett–Teller (BET) specific surface area of RHC was 33 m^2^ g^–1^, which is much higher than those of conventional carbonaceous active materials composed of graphite and hard carbon. The average pore width was calculated to be 9.7 nm from the total pore volume (0.08 m^2^ g^–1^), suggesting that free voids having an average width of ~ 10 nm (mesopore domain) exist in RHC. The X-ray diffraction (XRD) pattern of RHC exhibited two broad peaks at the approximate 2*θ* values of 23° and 43°. The former was attributed to the overlapping of disordered C and SiO_x_ structures and the latter was solely attributed to disordered C structures^44^, suggesting that RHC was composed of amorphous C and SiO_x_.

**Figure 2:**
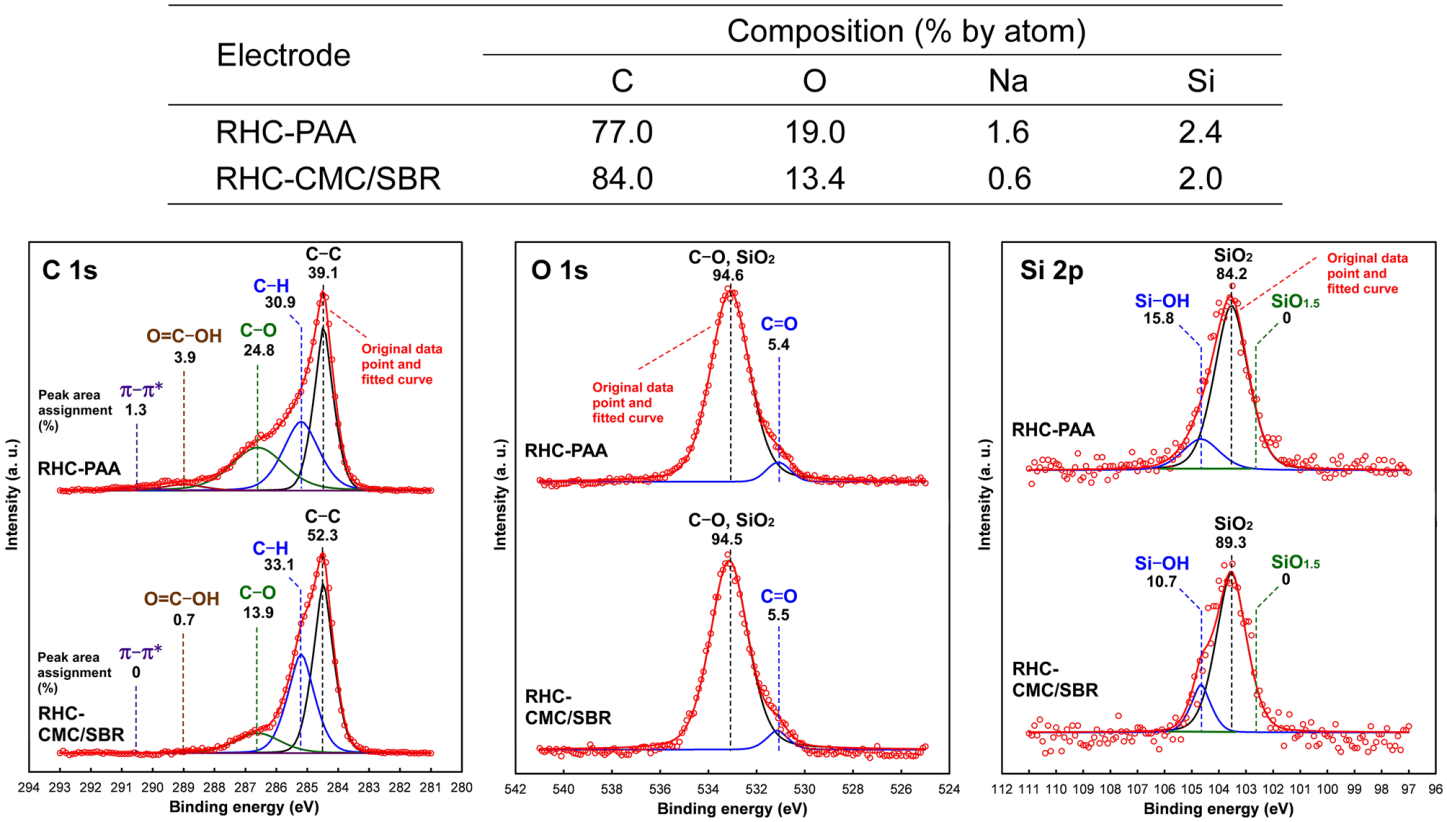
Atomic compositions calculated from the XPS survey spectra, and the C 1s, O 1s, and Si 2p spectra at the surfaces of pristine RHC-PAA and RHC-CMC/SBR electrodes. The percentage areas were assigned following the deconvolution of the peaks from the original C 1s, O 1s, and Si 2p spectra.

The chemical states of the fabricated RHC electrodes were investigated using X-ray photoelectron spectroscopy (XPS). The electrode fabricated using the PAA binder was labelled as RHC-PAA, while the one fabricated using CMC/SBR was labelled as RHC-CMC/SBR. Figure 2 shows the atomic compositions calculated from the wide scan spectra. The same figure also includes the narrow-scan spectra of C 1s, O 1s, and Si 2p at the surfaces of the pristine RHC-PAA and RHC-CMC/SBR electrodes. Increases in the fractions of O and Na and the resulting decrease in the fraction of C were observed on the RHC-PAA electrode. Additionally, 2% (by atom) of Si included in SiO_x_ was detected on both electrodes. The difference in the Si fractions of the RHC-CMC/SBR and RHC-PAA electrodes was insignificant, indicating that the RHC particles in both the electrodes were covered with binder layers of approximately equal thickness. The fraction of Na was derived from those of PAA and CMC in the form of sodium acetate. The narrow-scan spectra were deconvoluted into several peaks constituted of 70% Gaussian and 30% Lorentzian functions. These peaks were assigned to particular chemical bonds. For all the spectra, the area assignments of the separated peaks were calculated quantitatively. The C 1s spectra were separated into 5 peaks. These peaks represent components of C–C bonds derived from the graphene layer at 284.4 eV^47^; C–H bonds at 285.2 eV^48^; C–O bonds at 286.6 eV^48^, O=C–OH bonds at 289.0 eV ^49^; π–π* transition in aromatic rings at 290.5 eV^50^. The proportions of C–O and O=C–OH in RHC-PAA were found to be higher than those in RHC-CMC/SBR. The O 1s spectra were dominated by the peaks attributed to C–O and SiO_2_ at ~ 533 eV^35,51^, although weak satellite peaks attributed to C=O appeared at ~ 531 eV^35^. No significant difference was observed between the two O 1s spectra. The Si 2p spectra displayed the peaks attributed to SiO_2_ (Si(–O_4_)) at 103.5 eV^52^ and to Si–OH at 104.7 eV^53^. A peak attributed to SiO_1.5_ (Si(–O_3_)) can appear at 102.8 eV^52^, but it was negligible on both O 1s spectra. In the surface-layer of the RHC electrodes, x in SiO_x_ was approximately 2, and a higher proportion of Si–OH was observed in RHC-PAA.

### Rate behaviors of the RHC electrodes prior to complete lithiation

**Figure 3:**
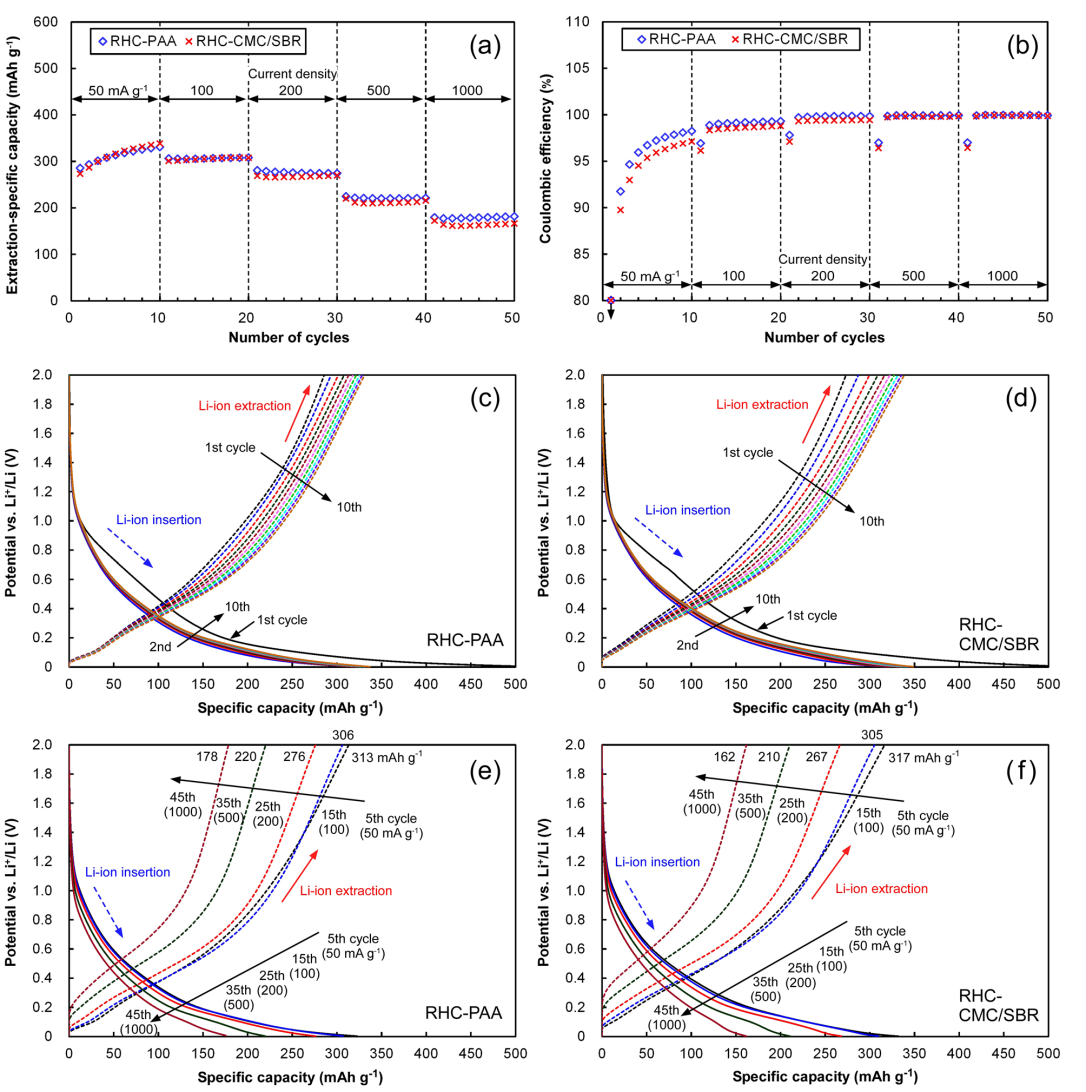
Li ion insertion/extraction performances of the RHC-PAA and RHC-CMC/SBR electrodes in the initial rate-test. (**a**) Li ion extraction-specific capacity, (**b**) coulombic efficiency of Li ion insertion/extraction, potential–specific capacity profiles of (**c**) RHC-PAA and (**d**) RHC-CMC/SBR electrodes during the initial cycles at 50 mA g^–1^, and potential–specific capacity profiles of (**e**) RHC-PAA and (**f**) RHC-CMC/SBR electrodes at different current densities.

Figure 3 plots the Li-ion insertion/extraction performances of the RHC-PAA and RHC-CMC/SBR electrodes throughout the initial rate-test. Both electrodes exhibited similar initial insertion- and extraction-specific capacities and coulombic efficiencies. The low initial coulombic efficiency of approximately 53% is peculiar to RH-derived carbonaceous active materials, which has been demonstrated elsewhere^42,44^. The PAA binder achieved ~ 3% higher initial coulombic efficiency, suggesting a distinct influence of the binder on the Li-ion insertion/extraction process in the RHC. The PAA binder facilitates the more efficient formation of solid electrolyte interphase (SEI) layers on the RHC particles. Both electrodes were subsequently subjected to insertion/extraction cycling at different current densities. Through the 10 initial cycles at 50 mA g^–1^, both electrodes exhibited gradual increases in their extraction-specific capacities and coulombic efficiencies. Although the specific capacities of the two electrodes were comparable, the RHC-PAA electrode maintained a higher efficiency. As the current density increased, the extraction-specific capacity of both electrodes decreased. Over the 30 cycles, the difference between their specific capacities was negligible. The two electrodes displayed similar potential–specific capacity profiles regardless of the current density. In the initial rate-test, the influence of the binder on the Li ion insertion/extraction mechanism of the RHC active materials was not strong. The gradual increases in the extraction-specific capacities and the coulombic efficiencies through the initial 10 cycles are attributed to capacity enhancements at potentials higher than 0.5 V vs. Li^+^/Li.

### Rate behaviors of the RHC electrodes following complete lithiation

**Figure 4:**
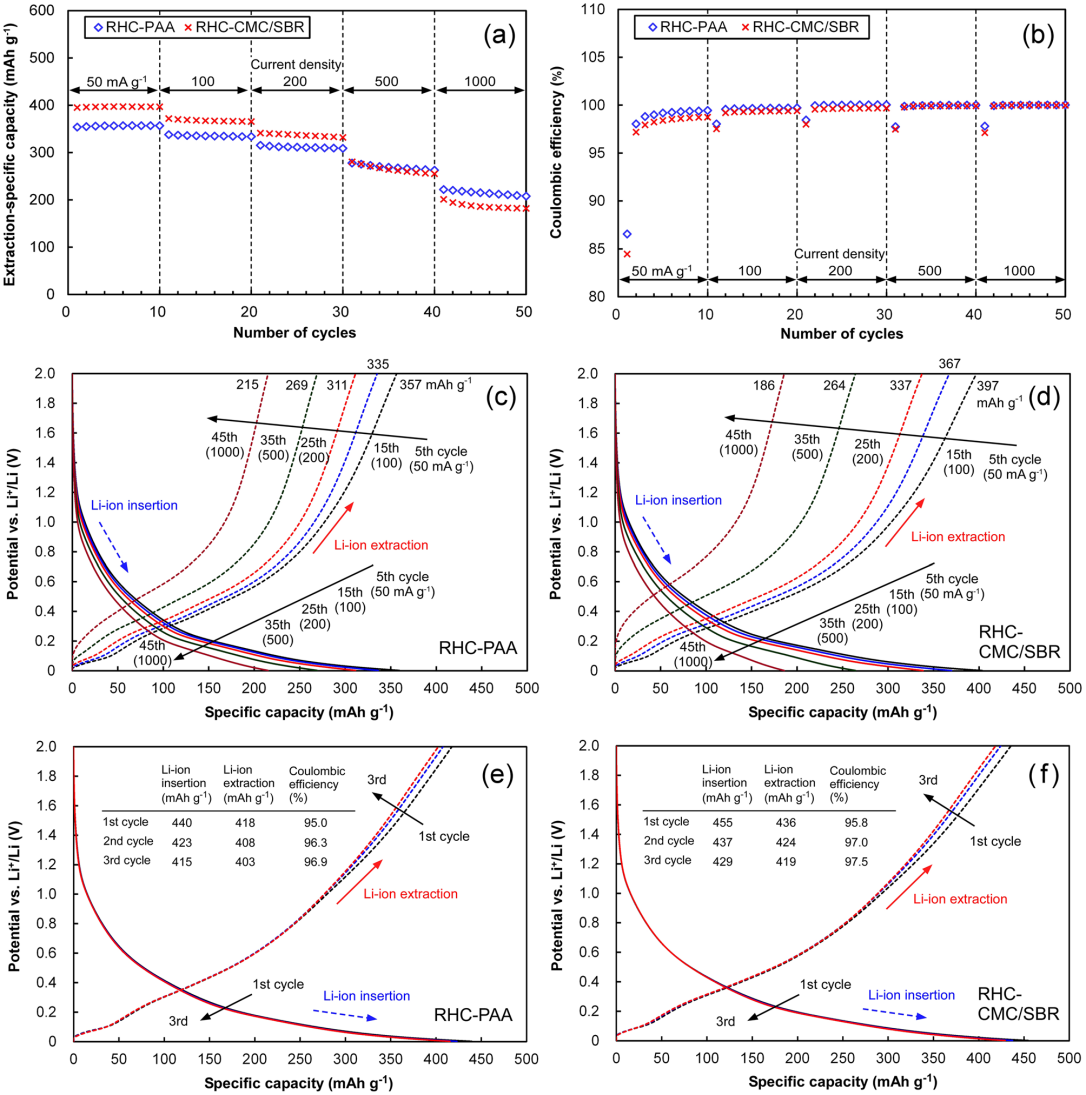
Li ion insertion/extraction performances of the RHC-PAA and RHC-CMC/SBR electrodes in the second rate-test, and those of the pristine electrodes. All the electrodes were completely lithiated (24 h-short) prior to the performance evaluation. (**a**) Li ion extraction-specific capacity, (**b**) coulombic efficiency of Li ion insertion/extraction, and potential–specific capacity profiles of (**c**) RHC-PAA and (**d**) RHC-CMC/SBR electrodes at different current densities. Potential–specific capacity profiles of pristine (**e**) RHC-PAA and (**f**) RHC-CMC/SBR electrodes following complete lithiation, during the three initial cycles at 50 mA g^–1^.

Following the initial rate-test, the cells were shorted to ensure the complete lithiation of RHC. The Li-ion insertion/extraction performances of the completely lithiated RHC-PAA and RHC-CMC/SBR electrodes were evaluated in a similar manner, and are plotted in Fig. 4. The performances of the pristine electrodes which were subjected to the complete lithiation process (24 h-cell short) but exempted from the rate test are also plotted therein. In contrast to the initial rate-test, neither sample exhibited a gradual increase in its specific capacity as the number of cycles increased. Following complete lithiation, a higher specific capacity for Li-ion extraction was exhibited by RHC-CMC/SBR than by RHC-PAA at current densities lower than 500 mA g^–1^. At the highest current density of 1000 mA g^–1^, RHC-PAA exhibited a higher specific capacity. The coulombic efficiency of RHC-CMC/SBR remained lower than that of RHC-PAA. A comparison to the initial rate-test clearly suggests that complete lithiation increased the specific capacity and the coulombic efficiency. The potential–specific capacity profiles reveal that the specific capacity at potentials lower than 0.2 V vs. Li^+^/Li increased due to complete lithiation, particularly at lower current densities. A higher specific capacity at lower potentials generally enhances the cell-voltage of LIBs. At the highest current density (1000 mA g^–1^), a larger IR drop was exhibited by RHC-CMC/SBR at the commencement of Li-ion extraction, indicating that more stable electrical networks were produced within the RHC-PAA electrode. Potential–specific capacity profiles of the pristine RHC-PAA and RHC-CMC/SBR electrodes recorded following complete lithiation indicate that higher specific capacity and coulombic efficiency were obtainable when lithiation was completed prior to Li-ion insertion/extraction cycling. Furthermore, the specific capacity decreased with the number of cycles. RHC-CMC/SBR exhibited higher specific capacity and coulombic efficiency during the three initial cycles than RHC-PAA did. This result suggests that pristine RHC-CMC/SBR is more porous than RHC-PAA, which facilitates the formation of a wider interface between the electrolyte and the RHC particles, and accelerates the lithiation processes, including SEI formation, SiO_x_ reduction, and Li passivation. Therefore, the pre-lithiation timing significantly influences the electrochemical performance of RHC. Following complete pre-lithiation, stability of the binder specie against the cycling of Li-ion insertion/extraction influenced the facilitation of Li-ion transport between the electrolyte and the bulks of the electrodes.

### Cycling stability of the RHC electrodes

**Figure 5:**
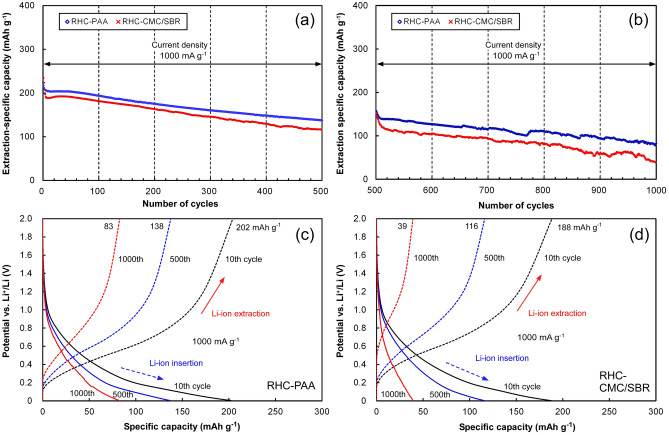
Li ion insertion/extraction performances of the RHC-PAA and RHC-CMC/SBR electrodes during the cycling test at 1000 mA g^–1^. The cycling test was suspended at the end of 500 cycles to acquire the impedance spectra, during which the electrode potential was maintained at 10 mV vs. Li^+^/Li for 1 h. (**a**) Li ion extraction-specific capacity as a function of number of cycles, (**b**) coulombic efficiency of Li ion insertion/extraction as a function of number of cycles, and potential–specific capacity profiles of (**c**) RHC-PAA and (**d**) CMC/SBR electrodes at different cycle numbers.

Cyclic Li-ion insertion/extraction was performed on the RHC electrodes subjected to the two rate-tests at a constant current density of 1000 mA g^–1^. The Li-ion extraction-specific capacities of the RHC-PAA and RHC-CMC/SBR electrodes and the potential–specific capacity curves are shown in Fig. 5. At the end of the 500 cycles, the cycling test was suspended for impedance analysis, during which time the electrodes were completely lithiated by maintaining their potential at 10 mV. Subsequently, the cycling test was restarted for 500 additional cycles. The initial specific capacity of both electrodes was ~ 200 mAh g^–1^. They exhibited similar gradual decreases in their specific capacities. RHC-PAA maintained a higher specific capacity throughout the cycling test. The difference between the specific capacities of RHC-PAA and RHC-CMC/SBR increased with the number of cycles. The retentions at the end of the cycling test were 41% for RHC-PAA and 21% for RHC-CMC/SBR, which were referred to as the specific capacities at the end of 10 cycles. The potential–specific capacity curves of the electrodes indicate that the loss of the specific capacity is primarily caused by a gradual decay of Li-ion uptake within the active materials. An increase in the IR drop was exhibited by both electrodes. RHC-CMC/SBR exhibited a considerable increase in its IR drop at the end of the cycling test.

### Impedance and morphological analysis

**Figure 6:**
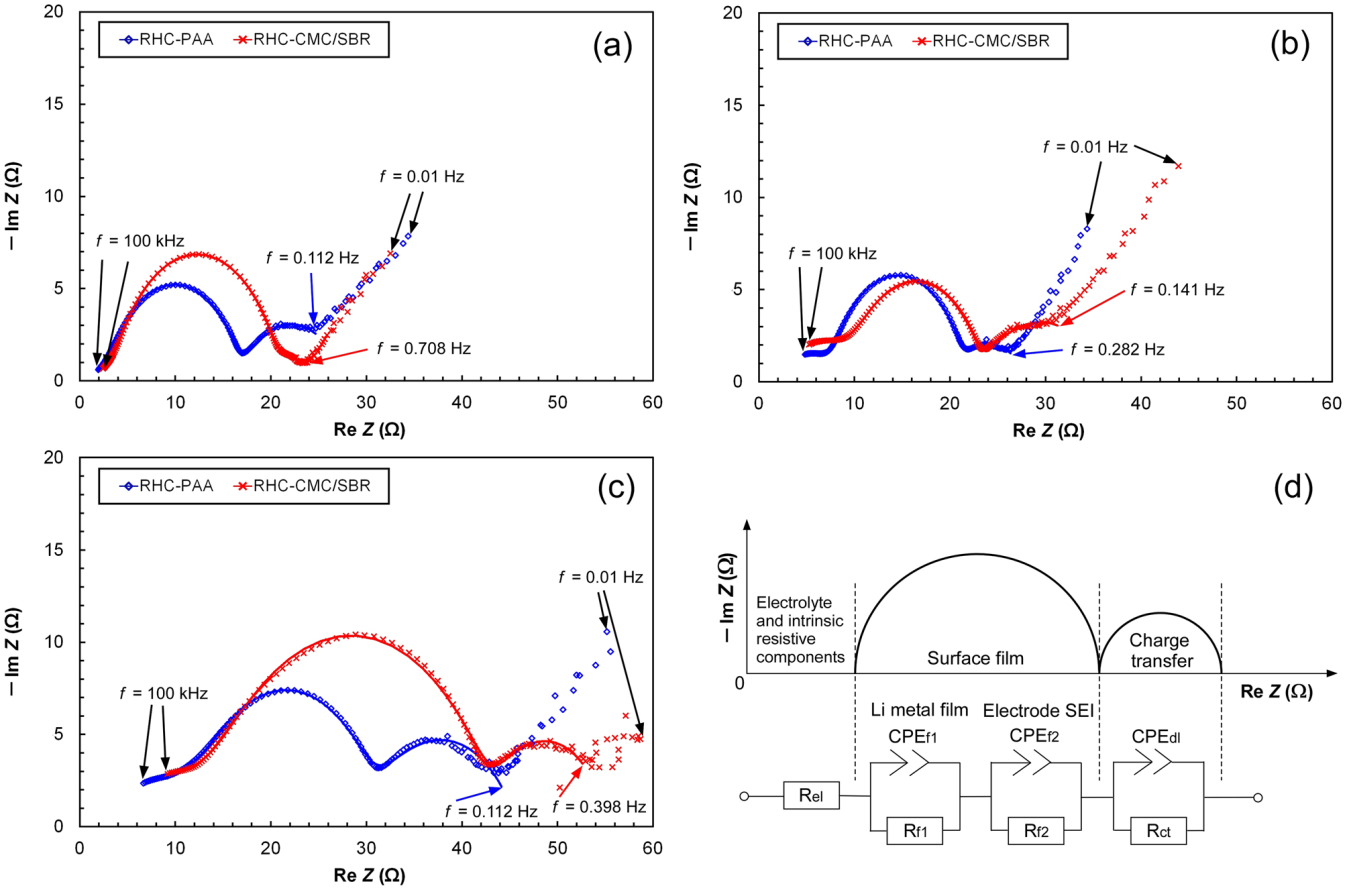
Impedance spectra (Nyquist plots) of the RHC-PAA and RHC-CMC/SBR electrodes in the cycling test, with the curves fitted using the equivalent circuit. The electrodes were biased at 10 mV vs. Li^+^/Li and subjected to AC voltage having an amplitude of 5 mV and frequency varying from 100 kHz to 10 mHz. (**a**) Before the cycling test, (**b**) after 500 cycles, (**c**) after 1000 cycles, (**d**) equivalent circuit representing the half-cell consisting of the RHC electrode and the counter electrode fabricated using Li metal.

Electrochemical impedance analysis was carried out for the RHC-PAA and RHC-CMC/SBR electrodes during the cycling test. Figure 6 plots their impedance spectra (Nyquist plots) with the fitted curves using the equivalent circuit representing the half-cell consisting of the RHC electrode and the counter electrode fabricated using Li metal. A component of the resistance is contributed by the electrolytic solution and the intrinsic resistance. In the equivalent circuit, this was expressed as the electrolytic solution resistance, R_el_. R_el_ possesses only the real part and is equivalent to the distance between the origin of the chart and the left-extremity of the capacitive semicircle in the higher frequency region. SEI films can be generated on the RHC electrode and the counter electrode fabricated using Li metal, because the Li ion shuttles between them. The SEI formation was responsible for one or two capacitive semicircles in the high-to middle-frequency region. The SEI films were electrochemically arranged as two parallel circuits: the constant phase element (CPE) and the resistor (R). A CPE is generally used to model the behavior of an imperfect capacitor, such as a non-homogeneous or porous electrode^54^. The impedance of the CPE (*Z*_CPE_) can be expressed using Eq. (1):

1 $$Z_{{{\text{CPE}}}} = \frac{1}{{\left( {{\text{j}}\omega } \right)^{p} T}}$$

where j is the imaginary unit, *ω* is the angular frequency, *p* is the exponent of CPE (0 < *p* ≤ 1), and *T* is its coefficient. The SEI film generated on the RHC electrode or the Li metal was represented by the parallel connection between CPE_f_ and R_f_. The electrode/electrolyte interface exhibits capacitive behavior because an electric double-layer is formed on the electrolyte side, whose capacitance is called the electric double-layer capacitance. This was shown by CPE_dl_. An interfacial potential difference applied to the double-layer produces a driving force for the charge-transfer reaction. The underlying resistance during the charge transfer, which is known as the charge-transfer resistance (R_ct_) and is an index of the difficulty of the charge transfer, was connected with CPE_dl_ in parallel. The bulk of the electrode included a resistive component preventing the diffusion of Li ions, which is called the Warburg impedance. It is represented by a straight line with a certain angular inclination with respect to the real-number axis in the low-frequency range^55^. Since the present study targets the electrode binders adhering to the surface of the active materials, the diffusion of Li ions in the electrode bulk, which is related to the Warburg impedance, was not considered. Only the semicircles appearing on the impedance spectra at frequencies greater than 0.1 Hz were analyzed. The circuit parameters were optimized using the software to fit the simulated data to the actual impedance spectra as closely as possible.

The impedance spectra of both RHC electrodes prior to cycling displayed a primary semicircle that appeared at the higher frequency (lower real part impedance) region, and a secondary semicircle which was smaller. The primary semicircle was overlapped by the two parallel connections of CPE_f_ and R_f_, which were caused by the surface films on the counter and RHC electrodes. The diameter of the real part of the primary semicircle for the RHC-PAA electrode was smaller than that of the RHC-CMC/SBR, implying that surface films possessing higher resistances were generated on the RHC-CMC/SBR electrode. The secondary semicircles were responsible for the charge-transfer mechanisms. A larger secondary semicircle was observed on the RHC-PAA electrode, indicating that its charge-transfer resistance was higher than that of the RHC-CMC/SBR electrode. Both RHC electrodes subjected to 500 cycles exhibited a separation of their primary semicircles. The diameter of the real part of the primary semicircles increased owing to the shuttling of the Li-ion between the counter and RHC electrodes. The RHC-CMC/SBR electrode exhibited an increase in the diameter of the secondary semicircle related to the charge-transfer mechanism. At the end of 1000 cycles, the primary and secondary semicircles were found to be enlarged for both electrodes. The diameter of the primary semicircle of the RHC-CMC/SBR increased greatly through the final 500 cycles. The separated semicircle appearing in the spectra of the RHC-CMC/SBR at higher frequencies (near the origin of the chart) was small, suggesting that a highly-resistive and thick SEI film was formed on the RHC-CMC/SBR electrode following the cycling.

The circuit parameters determined are listed in Table 1. The *R*_el_ of neither electrode changed appreciably following the initial 500 cycles (1.1–1.4 Ω). However, the RHC-PAA and RHC-CMC/SBR electrodes subjected to 1000 cycles displayed lower (0.6 Ω) and negligible *R*_el_, respectively. The impedance data obtained at the highest frequency (100 kHz) were distant from the imaginary axis. In these cases, it appears to be impossible to individually and accurately calculate *R*_el_ and *R*_f1_, although it is possible to obtain their sum. The contribution of *R*_el_ to the total resistance, defined as *R*_all_ = *R*_el_ + *R*_f1_ + *R*_f2_ + *R*_ct_, was insignificant. Both RHC electrodes exhibited large increases in *R*_f1_ and *R*_f2_ instead of *R*_ct_ because of the cyclic insertion and extraction of the Li ion. The surface films on both electrodes increased the resistance. In particular, the resistance from the SEI film on RHC-CMC/SBR increased significantly between cycles 501 and 1000.

**Table 1:** Impedance fitting parameters for the RHC-PAA and RHC-CMC/SBR electrodes.

	Circuit element	R_el_	CPE_f1_		R_f1_	CPE_f2_		R_f2_	CPE_dl_		R_ct_	R_all_
	Parameters	*R*_el_ (Ω)	*p*_f1_	*T*_f1_ (Fs^(p–1)^)	*R*_f1_ (Ω)	*p*_f2_	*T*_f2_ (Fs^(p–1)^)	*R*_f2_ (Ω)	*p*_dl_	*T*_dl_ (Fs^(p–1)^)	*R*_ct_ (Ω)	*R*_all_ (Ω)
RHC-PAA	Before cycling test	1.07	0.460	1.69 × 10^–3^	3.04	0.830	1.10 × 10^–4^	12.7	0.640	5.41 × 10^–2^	11.0	27.7
	500 cycles later	1.39	0.488	2.23 × 10^–4^	7.12	0.897	9.28 × 10^–5^	12.5	0.619	4.20 × 10^–2^	7.13	28.2
	1000 cycles later	0.638	0.366	7.58 × 10^–4^	16.3	0.869	9.47 × 10^–5^	14.4	0.692	1.75 × 10^–2^	14.4	45.8
RHC-CMC/SBR	Before cycling test	1.36	0.436	1.84 × 10^–3^	3.61	0.874	6.67 × 10^–5^	15.6	0.628	4.41 × 10^–2^	3.71	24.3
	500 cycles later	1.12	0.478	2.33 × 10^–4^	10.3	0.873	1.08 × 10^–4^	11.5	0.563	5.26 × 10^–2^	13.5	36.4
	1000 cycles later	1.00 × 10^–5^	0.367	3.87 × 10^–4^	17.1	0.782	8.14 × 10^–5^	26.5	0.802	1.71 × 10^–2^	11.7	55.2

*R*_all_ = *R*_el_ + *R*_f1_ + *R*_f2_ + *R*_ct_.

**Figure 7:**
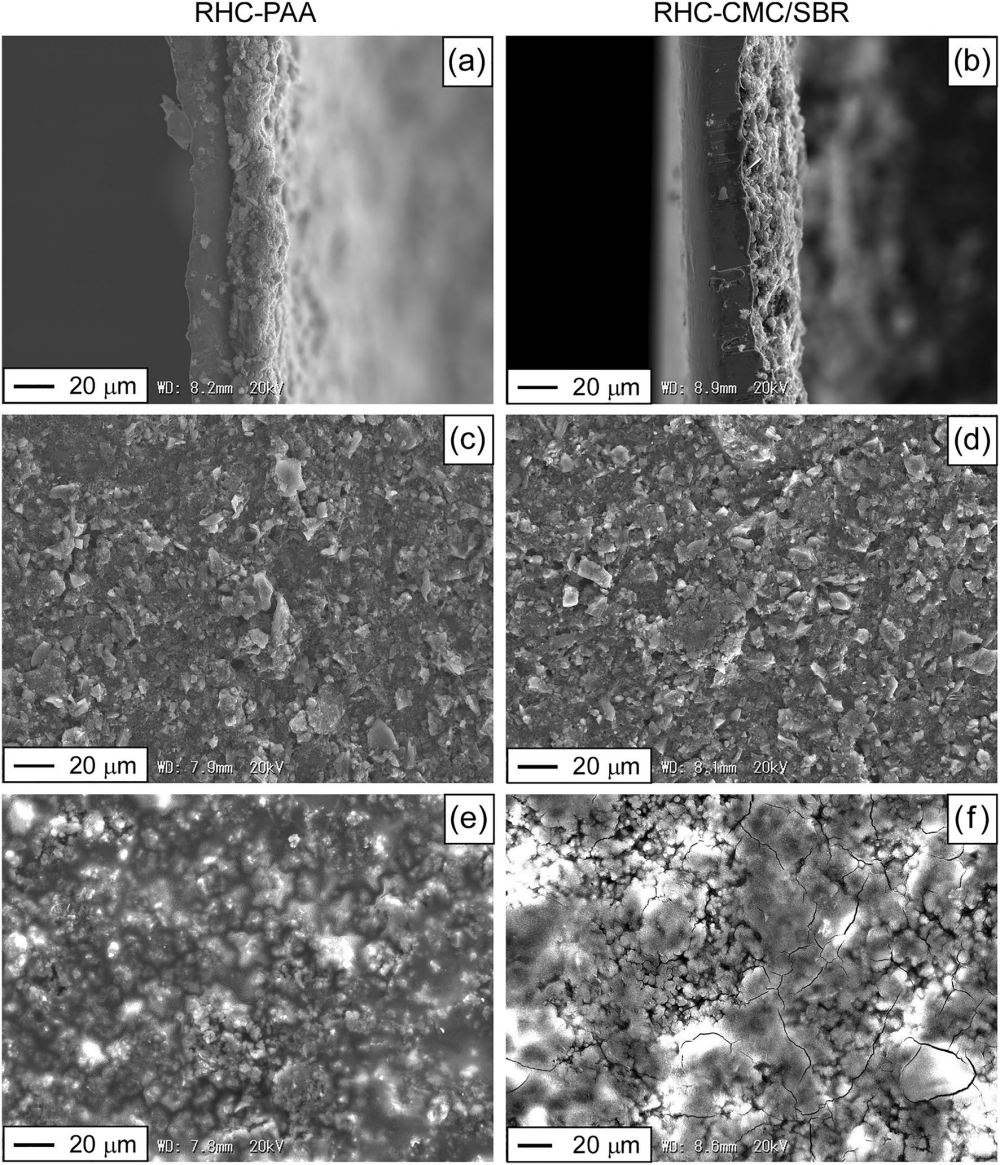
Microscopic morphologies of the RHC-PAA and RHC-CMC/SBR electrodes. Cross-section of pristine (**a**) RHC-PAA and (**b**) RHC-CMC/SBR electrodes, surface of (**c**) pristine RHC-PAA, (**d**) pristine RHC-CMC/SBR, (**e**) 1000-times cycled RHC-PAA, and (**f**) 1000-times cycled RHC-CMC-SBR electrodes.

Figure 7 shows the microscopic morphologies of the RHC electrodes. The cross-sectional views of the pristine RHC electrodes indicated that in the RHC-PAA electrode, the RHC active material was robustly coated by the conductive agent on the Cu current collectors, whereas a slight landslide was observed at the edge of the RHC-CMC/SBR electrode. The SEM surface views of the pristine electrodes indicated that the internal structure of RHC-CMC/SBR was more porous. Thick SEI deposition derived from lithiated byproducts was observed on both electrodes aged through 1000 cycles. The RHC-CMC/SBR electrode exhibited thicker SEI formation, which was consistent with the noticeable increase in its *R*_f2_.

### Role of the aqueous binder on the anodic performance of the RHC electrode

The coulombic efficiencies of the RHC-PAA and RHC-CMC/SBR electrodes during the initial Li-ion insertion/extraction were 54.9 and 51.7%, respectively. Both electrodes exhibited gradual increases in their extraction-specific capacity and coulombic efficiency through the initial 10 cycles. The low and unstable coulombic efficiency exhibited through the initial Li-ion insertion/extraction cycles implies that RHC possesses a large and volatile irreversible capacity, which is related to the formation of SEI, reduction of silicon oxides, and passivation of Li. Following complete lithiation, both electrodes displayed a stable specific capacity and a high coulombic efficiency. These results clearly suggest that RHC electrodes require a pre-lithiation process prior to actual anodic application in LIBs^56^.

Following complete lithiation, RHC-CMC/SBR exhibited higher specific capacities at current densities lower than 500 mA g^–1^, whereas RHC-PAA exhibited a higher specific capacity at the highest current density (1000 mA g^–1^) and during the cycling test. The specific capacity retentions of RHC-PAA and RHC-CMC/SBR at the end of the cycling test were 41 and 21%, respectively. The IR drops, which serve as an indication of the internal resistance of the electrode, were smaller for RHC-PAA when subjected to the highest current density. The impedance analysis suggests that the increase in the electrode resistance occurs due to the formation of SEI films, and RHC-CMC/SBR exhibited a significant increase in resistance.

The C 1s peak assignments in XPS indicate that the proportions of C–O and O=C–OH in RHC-PAA (28.7%) are significantly higher than those in RHC-CMC/SBR (14.6%). The Si 2p spectra demonstrate the formation of SiO_2_ and Si–OH on the surfaces of both RHC electrodes. The ester-like bonds of the aqueous binders help to stabilize the electrode structure and thereby improve the electrochemical performance of SiO-based LIB anodes^17,30^. The C–O and O=C–OH bonds of the electrode binders indicate hydroxyl groups that have a high affinity for SiO_2_ and Si–OH at the surface of SiO_x_. More tenacious adhesion between RHC particles, and between RHC and conductive agent particles, were produced within the pristine RHC-PAA than the pristine RHC-CMC/SBR, which is verified by their SEM cross-sectional and surface views. Therefore, the PAA binder strengthens the electrode, which is responsible for its ability to maintain its higher specific capacity at high current densities. In addition, the amorphous structure of PAA may be useful for tightly binding and covering the active material particles^57^. However, the more porous structure of the RHC-CMC/SBR may enhance the surface area of RHC particles that are in contact with the electrolyte to produce wider SEI films. Therefore, RHC-CMC/SBR delivered higher specific capacity than RHC-PAA irrespective of whether its complete lithiation was performed before or after the cycling of Li-ion insertion/extraction. Although the RHC-CMC/SBR sample that was subjected to complete lithiation but exempted from cycling delivered higher coulombic efficiency, RHC-PAA exhibited greater structural stability against the cycling of Li-ion insertion/extraction as well as higher coulombic efficiency and superior cycling performance during the initial and second rate tests. The stability of the binder specie against cycling degradation influenced the facilitation of Li-ion transport into the RHC particles.

## Conclusions

The Li-ion insertion and extraction properties of RHC electrodes fabricated using different aqueous binders, namely PAA and CMC/SBR, were investigated for their anodic application in LIBs. The low and unstable coulombic efficiencies through the initial Li-ion insertion/extraction cycles of both RHC-PAA and RHC-CMC/SBR electrodes indicated that RHC requires a pre-lithiation process prior to actual anodic application. Therefore, the rate and cycling performances of completely lithiated RHC electrodes were evaluated in a half-cell configuration. The impedance spectra of the RHC electrodes were acquired before and after the cycling tests to identify the source of the increase in electrode resistance. The high affinity of PAA for the SiO_x_ component in RHC strengthened the electrode. This electrode maintained a lower resistance and restricted the enhancement of the RHC surface area to produce SEI films. Therefore, the completely lithiated RHC-PAA displayed a higher, stable specific capacity for the insertion/extraction of the Li ion at the highest current density (1000 mA g^–1^) and during the 1000-cycle test. The internal structure of RHC-CMC/SBR was more brittle and porous; therefore, it possessed higher specific capacity at current densities lower than 500 mA g^–1^, but exhibited a larger and more volatile irreversible capacity. Finally, it was concluded that the PAA binder was more suitable for the RHC electrode which essentially needs a pre-lithiation process prior to use in LIB anodes.

## Methods

### Production of RHC

RH obtained by de-husking Japanese paddy rice was supplied from a rice farmer in Senboku City, Akita Prefecture, Japan, as the experimental material with his permission, and was pre-carbonized in a tubular furnace under nitrogen gas flow (1 L/min). RH was heated to 600 °C in 1 h, maintained at 600 °C for 1 h, and thereafter naturally cooled to 25 °C. The pre-carbonized RH was washed using distilled water to remove impurities until the pH of the washing drain became lower than 9.0. The washed RH was dried in an oven at 100 °C for 12 h. For further carbonization, the washed pre-carbonized RH was heated to 1000 °C under nitrogen flow in 1 h, maintained at 1000 °C for 1 h, and thereafter naturally cooled to room temperature and milled using a planetary ball mill at a rotational speed of 400 rpm for 5 min, producing powdered RHC.

### Fabrication of RHC electrodes

Two species of electrode binders, PAA (AQUACHARGE, Sumitomo Seika Chemicals Co., Ltd., Japan) and a mixture of CMC (Cellogen 7A, DKS Co. Ltd., Japan) and SBR (TPD 2001, JSR Corp., Japan) were used for the fabrication of RHC electrodes. Acetylene black (DENKA BLACK, Denka Co. Ltd., Japan) was used as the conductive agent. The slurry was prepared to coat the RHC active material using an applicator on the current collector, which was fabricated using Cu foil (t20 μm, no surface treatment, Hohsen Corp., Japan). The slurry was composed of 85% (w/w) RHC, 10% (w/w) acetylene black, and 5% (w/w) binder. To fabricate the RHC-CMC/SBR electrode, a binary binder composed of 2.5% (w/w) CMC and 2.5% (w/w) SBR was used. The coated Cu foil was dried at 100 °C in air for longer than 6 h, and subsequently perpendicularly pressed at a pressure of 2 MPa and punched into *⌀*15 mm disks. The punched disks were used as the sample electrodes. The mass loading of the active material and the thickness of the coating on the electrodes were approximately 4 mg and 35 μm, respectively.

### Characterization of RHC and RHC electrodes

Thermogravimetric analysis was performed in the range of 140–850 °C at a heating rate of 10 °C/min in air flow (200 mL min^–1^) using a thermogravimetric analyzer (Thermo Plus Evo, Rigaku Corp., Japan). To exclude the effect of residual moisture in RHC on its thermogravimetric response, the mass at 140 °C was selected as the reference value (100%). The particle size distribution of RHC was ascertained using a laser-diffraction-type particle size distribution analyzer (SALD-200 V, Shimadzu Corp., Japan), providing a mean diameter for the RHC particles. The pore structure of RHC was evaluated by plotting a nitrogen adsorption–desorption isotherm at – 196 °C using a gas adsorption analyzer (Autosorb-3B, Quantachrome Instruments Inc., USA). The Brunauer–Emmett–Teller (BET) specific surface area was calculated based on the BET theory. The volume of nitrogen adsorbed at a relative pressure of 0.98 was taken as a representation of the total pore volume. The average pore width was calculated from the BET specific surface area and the total pore volume. X-ray diffraction (XRD) patterns were recorded on a diffractometer (RINT-2020 V, Rigaku Corp., Japan) using Cu-Kα radiation (wavelength: 0.15418 nm) to evaluate the crystallinity of RHC. The tube voltage and current were 40 kV and 40 mA, respectively.

X-ray photoelectron spectroscopy (XPS) was performed to investigate the chemical states of the fabricated RHC electrodes. An X-ray photoelectron spectroscope (AXIS Ultra DLD, Kratos Analytical Ltd., UK) was used to obtain wide- (survey) and narrow-scan spectra, by employing Al Kα as the radiation source (135 W). Wide-scan spectra were acquired at the binding energy of 1200–0 eV with the pass energy set at 160 eV. The atomic composition at the surface of the RHC electrodes was calculated based on the area of the targeted peaks (C 1s, O 1s, Na 1s, and Si 2p) using the included software. The narrow-scan spectra of C 1s, O 1s, and Si 2p were collected at a pass energy of 20 eV and analyzed in detail using XPS peak-separation software (COMPRO12, The Surface Analysis Society of Japan, Japan). The electrodes that were dried at 140 °C in vacuum for longer than 6 h and cooled to 25 °C were introduced into the spectroscope. The binding energy of the spectrum was corrected on the basis of the C 1s peak at 284.5 eV^58^. Scanning-electron microscopy (SEM, VE-8800, Keyence Corp., Japan) was employed to observe the morphology of the RHC electrodes before and after the electrochemical tests.

### Cell assembly

Half-cells incorporating the RHC electrode and Li foil as the counter electrode were assembled using a 2-electrode cell fabricated using SUS 304 steel (HS cell, Hohsen Corp., Japan). A piece of propylene membrane separator (⌀23 mm, 2500, Celgard LLC, USA) was sandwiched between the Li foil (⌀15 mm, t0.2 mm, Honjo Metal Co., Ltd., Japan) and the RHC electrode, which was previously dried at 140 °C in vacuum for longer than 6 h and subsequently cooled to 25 °C. One milliliter of the LIB electrolyte, which was a mixed solution of ethylene carbonate and diethyl carbonate (1:1 by vol.) containing LiPF_6_ at a concentration of 1 mol/L (Kishida Chemical Co., Ltd., Japan) was injected into the half-cell. The half-cells were assembled in a glove box filled with pure argon gas.

### Electrochemical tests

The Li-ion insertion and extraction behaviors of the electrodes prepared were observed by decreasing the potential from 2 to 0.002 V vs. Li^+^/Li and by increasing it from 0.002 to 2 V vs. Li^+^/Li, respectively. The density of current passing through the half-cell was increased in a stepwise manner through 50, 100, 200, 500 and 1000 mA g^–1^. Current density is defined as the total current divided by the mass of the active material (RHC). The Li-ion insertion/extraction process was repeated 10 times at each current density, totaling 50 cycles of Li-ion insertion/extraction. This sequence was defined as the initial rate-test. A battery charge–discharge system (HJ1005SD8, Hokuto Denko Corp., Japan) was used for the measurements. The specific capacity for Li-ion insertion or extraction (in mAh g^–1^) was defined as the time-integral of current (electric charge) divided by the active mass. The coulombic efficiency was calculated by dividing the extraction-specific capacity by the insertion-specific capacity. Following this sequence, the cell was shorted for 24 h to completely saturate the RHC with Li ions, so as to completely eliminate the irreversible capacity resulting from the formation of a SEI, reduction of SiO_x_, and passivation of Li, which is defined as the complete lithiation process. Thereafter, the cell was subjected to the second rate-test, for which the test conditions were similar to those for the initial rate-test. Subsequently, a cycling test was conducted. The Li-ion insertion/extraction process was repeated 1000 times at a potential range of 0.002–2 V vs. Li^+^/Li and a current density of 1000 mA g^–1^. The Li-ion insertion/extraction performance of the pristine electrode that was subjected to the complete lithiation process (cell short for 24 h) but exempted from the rate test was separately evaluated to investigate the influence of the lithiation timing on the electrochemical performance of RHC.

Impedance analysis was performed before and after the cycling test, and at the end of 500 cycles, using an electrochemical measurement system (HZ-5000, Hokuto Denko Corp., Japan). A sinusoidal voltage having a frequency in the range of 100 kHz to 10 mHz and an amplitude of 5 mV was applied to the cell in combination with a DC bias voltage of 10 mV to obtain a Nyquist plot, providing the impedance information of the sufficiently-lithiated RHC electrodes. The cell voltage was maintained at 10 mV for 15 min prior to the application of the sinusoidal voltage. Impedance analysis required 50 min. Based on the shape of the Nyquist plot obtained, an equivalent circuit model was proposed. With the aid of software installed on the aforementioned measurement system (EIS Version 1.0.14, Hokuto Denko Corp., Japan), the parameters of the elements embedded in the proposed equivalent circuit were optimized to fit the Nyquist plot obtained as closely as possible to the one simulated. All the above electrochemical tests were carried out at 25 °C.
